# Multidimensional sleep behaviours and incident obesity in postmenopausal women: a prospective cohort study

**DOI:** 10.3389/fendo.2026.1890100

**Published:** 2026-08-17

**Authors:** Ming Zhu, Siyuan Liu, Xiaokun Gang, Yao Wang

**Affiliations:** 1 Department of Endocrinology and Metabolism, The First Hospital of Jilin University, Changchun, China; 2 Department of Orthopedics , The Second Hospital Jilin University, Changchun, China

**Keywords:** mediation analysis, metabolomics, obesity, postmenopausal women, prospective cohort study, sleep behaviors

## Abstract

**Objective:**

Postmenopausal women are at high risk of both sleep disorders and obesity, yet prospective evidence linking multidimensional sleep behaviors to obesity risk in this population remains limited. This study aimed to examine the prospective associations between multidimensional sleep behaviours and incident obesity in postmenopausal women, and to explore whether blood biomarkers and plasma metabolites provide exploratory biological evidence.

**Methods:**

This prospective cohort study included 68, 419 non-obese postmenopausal women from the UK Biobank. Sleep behaviors (duration, insomnia, chronotype, snoring, daytime sleepiness) were collected via questionnaires. Obesity events were identified using ICD-10 codes. Cox proportional hazards models were used to assess associations, and exploratory indirect-association analyses were performed to evaluate the potential involvement of blood biomarkers and plasma metabolites.

**Results:**

During a median follow-up of 13.33 years, 1, 146 incident obesity cases were documented. Sleep duration showed an approximately U-shaped association with obesity risk: short sleep (≤6 hours, HR = 1.214) and long sleep (≥10 hours, HR = 1.654) were both associated with increased risk. Frequent insomnia (HR = 1.320), evening chronotype (HR = 1.224), and snoring (HR = 1.234) were also independent risk factors. Exploratory indirect-association analyses suggested small signals involving inflammation- and lipid-related biomarkers, but these findings should be interpreted as hypothesis-generating.

**Conclusion:**

Abnormal sleep duration, frequent insomnia, evening chronotype, and snoring were associated with a higher rate of incident obesity in postmenopausal women. Exploratory biomarker and metabolomic analyses provided only hypothesis-generating biological evidence. Incorporating sleep health management into obesity prevention strategies for this population is warranted.

## 1 Introduction

Globally, obesity has become a serious public health problem affecting more than 2 billion people and significantly increasing the risk of type 2 diabetes mellitus (T2DM), cardiovascular disease (CVD), metabolic dysfunction-associated fatty liver disease (MASLD), and multiple cancers (1). The occurrence of obesity involves a complex pathophysiological mechanism, which has not yet been fully elucidated. Therefore, it is of great significance to identify more risk factors that can be intervened for the prevention and management of obesity.

Adequate sleep forms the foundation for maintaining physiological, psychological, and emotional health, and has been demonstrated to play a vital role in regulating energy balance and metabolic homeostasis. The National Sleep Foundation recommends that adults ensure 7–9 hours of sleep per night to maintain optimal functional status. Insufficient or excessive sleep may have adverse effects on health (2). Previous studies have revealed that multidimensional sleep behaviour abnormalities—including abnormal sleep duration, insomnia, circadian rhythm disruption, snoring and daytime sleepiness—are closely associated with elevated obesity risk (3–6).

It is noteworthy that postmenopausal women constitute a dual-risk group for sleep disorders and obesity. On the one hand, approximately 40%–60% of perimenopausal and postmenopausal women experience varying degrees of sleep issues, commonly manifesting as fragmented sleep, increased nocturnal awakenings and diminished sleep quality (7). On the other hand, the abrupt decline in oestrogen levels following menopause leads to increased body fat and redistribution of visceral fat, further exacerbating the risk of obesity and metabolic disorders (8–10). These characteristics suggest that poor sleep behaviour may play a particularly important role in the occurrence of obesity in this population. However, although the association between sleep and obesity has been extensively documented in the general population, research specifically targeting postmenopausal women remains relatively limited. The specific endocrine changes, body composition changes and sleep pattern changes in this population may make the mechanism of sleep behaviour on obesity different from that of the general population, but it has not been systematically evaluated. Consequently, a thorough investigation into the relationship between sleep characteristics and obesity risk in postmenopausal women would not only help elucidate the underlying causes of obesity in this group but also provide crucial evidence for developing targeted prevention strategies. We prespecified the hypothesis that abnormal multidimensional sleep behaviours would be prospectively associated with a higher incidence of obesity among postmenopausal women. This study was designed as an explanatory, association-based analysis rather than a prediction model or causal-mechanistic study. Using UK Biobank data, we examined the associations of sleep duration, insomnia, chronotype, snoring, and daytime sleepiness with incident obesity, and explored blood biomarkers and plasma metabolites might offer exploratory biological clues, aiming to provide evidence for the early clinical identification of postmenopausal women at high risk of obesity and to formulate personalised sleep intervention strategies for them.

## 2 Methods

### 2.1 Study population

This study was based on the UK Biobank cohort, which recruited more than 500, 000 adults aged 37–73 years living in the UK between 2006 and 2010. At enrolment, all participants completed detailed demographic, lifestyle and health-related questionnaires and provided blood and other biological samples. This study was approved by the North West Multi-Centre Research Ethics Committee (approval number 11/NW/0382), and all participants provided written informed consent. The data used in this study were obtained through the UK Biobank application (ID 511414). From 502, 129 UK Biobank participants, we sequentially excluded men (n = 228, 973), participants aged <40 years (n = 1), women who had not reached menopause or had unclear menopausal status (n = 63, 971), those with a history of ovarian or uterine resection (n = 20, 219), participants with implausible sleep duration (<3 or >20 h/day; n = 1, 151) and baseline BMI <18.5 or ≥30 kg/m² (n = 33, 186). We further excluded participants with negative follow-up time for ICD-coded obesity (n = 44) to avoid counting pre-baseline obesity records as incident events. We then excluded that missing covariate data (n = 74, 807) and missing sleep-related variables (n = 11, 358). The final analytic cohort included 68, 419 eligible non-obese postmenopausal women. The population screening process is detailed in **Supplementary Figure S1**.

### 2.2 Study variables

Sleep behaviours were collected via a touchscreen questionnaire (**Table S1**), encompassing five domains: sleep duration, chronotype, insomnia, snoring, and excessive daytime sleepiness. Sleep duration is measured by asking:”About how many hours of sleep do you get in every 24 hours?” The sleep duration was further divided into normal sleep group (7-9h), short sleep group (≤ 6h), and long sleep group (≥ 10h). Chronotype was assessed by asking: “Do you consider yourself to be (1) definitely a ‘morning’ person, or more a ‘morning’ than ‘evening’ person (2), definitely an ‘evening’ person or more an ‘evening’ than ‘morning’ person.” Insomnia symptoms were assessed by asking: ‘Do you have trouble falling asleep at night or do you wake up in the middle of the night?’, with response options classified as never/rarely, sometimes, and usually. Information on snoring was collected by asking: “Does your partner or a close relative or friend complain about your snoring?”, with response options of (1) yes or (2) no. Daytime subjective sleepiness was assessed by the question: “How likely are you to doze off or fall asleep during the daytime when you don’t mean to? (e.g., when working, reading, or driving)”, with response options classified as never/rarely, sometimes, and usually.

The primary outcome was ICD-coded incident obesity, defined using ICD-10 codes E66.0, E66.8, and E66.9 from hospital admission and death-registry records. This endpoint therefore reflects clinically recorded obesity rather than all BMI-defined obesity. The event date was defined as the first recorded date of the relevant ICD-10 code.

### 2.3 Covariates

All participants completed touchscreen questionnaires and anthropometric assessments at recruitment. Covariates included age, ethnicity, education score, Townsend Deprivation Index, smoking and drinking status, exercise metabolic equivalents, healthy diet score, diabetes, hypertension, number of live births, age at menarche, pregnancy loss or termination, oral contraceptive use, and hormone replacement therapy. Continuous and categorical definitions are detailed in **Supplementary Table S1**. All covariates were specified *a priori* based on existing knowledge because they may be associated with both sleep behaviours and clinically recorded obesity. Models were intended for association estimation rather than causal total-effect estimation. Diabetes and hypertension were included as baseline medical-history indicators of cardiometabolic health and potential healthcare contact.

### 2.4 Mediating variables

Mediating variables included standardised blood biochemical parameters (white blood cell count, neutrophil count, lymphocyte count, monocyte count, platelet count, red cell distribution width (RDW), mean platelet volume (MPV), plateletcrit (PCT), platelet distribution width (PDW), C-reactive protein (CRP), triglycerides, total cholesterol, low-density lipoprotein (LDL), high-density lipoprotein (HDL), Apolipoprotein A, Apolipoprotein B, Lipoprotein A, Glycated haemoglobin, Fasting blood glucose and Uric acid), alongside plasma metabolic markers derived from the high-throughput Nuclear Magnetic Resonance (NMR) analysis platform developed by Nightingale Health. The actual sample sizes for plasma metabolomic indirect-association analyses varied by sleep phenotype and metabolite availability and are summarised in **Supplementary Table S24**. Oestradiol was not retained in the exploratory indirect-association analyses because of extensive missingness and limited usable information among postmenopausal women. This study measured metabolic biomarkers in baseline EDTA plasma samples from approximately 275, 000 randomly selected participants in the UK Biobank. Each EDTA plasma sample was quantified for 249 metabolic indicators, comprising 168 absolute level measurements and 81 ratio measurements. These indicators included: detailed cholesterol metabolism parameters, fatty acid composition, and multiple low-molecular-weight metabolites such as amino acids, ketone bodies, and glycolytic metabolites. Further details are available at: https://biobank.ndph.ox.ac.uk/showcase/refer.cgi?id=3000 (accessed 11 June 2025).

### 2.5 Statistical analysis methods

According to whether postmenopausal women were obese, the subjects were divided into “participants who developed ICD-coded obesity during follow-up” and “participants who did not develop ICD-coded obesity during follow-up” for descriptive analysis. Categorical variables were expressed as frequencies (percentages), while continuous variables were presented as mean ± standard deviation (SD).

We employed Cox proportional hazards models to assess the association between multiple sleep behaviours (sleep duration, chronotype, snoring, insomnia symptoms, and daytime sleepiness) and the risk of obesity in postmenopausal women. The corresponding hazard ratio (HR) and 95% confidence interval (CI) were reported, and a multiple adjustment model was constructed to adjust for the potential confounding factors. Model 1 was adjusted for age and ethnicity. Model 2 was additionally adjusted for educational score, Townsend Deprivation Index, smoking status, alcohol drinking status, MET score, healthy diet score, diabetes, and hypertension. Model 3 was further adjusted for number of live births, age at menarche, pregnancy-loss history, oral contraceptive use, and HRT use. The primary prespecified analyses were the associations between five baseline sleep behaviours and ICD-coded incident obesity in the fully adjusted Cox model. Restricted cubic spline analysis, stratified analyses, formal interaction tests, sensitivity analyses, and biomarker/metabolomic indirect-association analyses were considered secondary or exploratory. In all models, we check the collinearity of covariates by the correlation matrix and the variance inflation factor (VIF), and the results show no multicollinearity. The proportional hazards assumption was assessed using Schoenfeld residuals.

A restricted cubic splines (RCS) model was used to explore the potential nonlinear relationship between sleep behaviour and obesity risk. Stratified analyses were conducted by age group, baseline BMI category, ethnicity, TDI tertile, HRT use, diabetes history, and hypertension history. To formally assess potential effect modification, interaction terms between each sleep behaviour and each subgroup variable were added to the fully adjusted model. These interaction tests were considered exploratory and were used to guide interpretation of stratified estimates.

Exploratory indirect-association analyses were performed using baseline sleep behaviours as exposures, blood biomarkers or plasma metabolites as continuous candidate intermediate variables, and ICD-coded obesity status during follow-up as a binary outcome. The candidate intermediate variables were measured at baseline, contemporaneously with sleep behaviours. The reported average indirect effect, average direct effect, total effect, and proportion were estimated on an additive probability-difference scale rather than the Cox hazard-ratio scale. Benjamini–Hochberg FDR correction was applied separately within each sleep phenotype across candidate blood biomarkers and, separately, across plasma metabolites. These analyses were interpreted as exploratory biological clues and were not used to infer causal mediation.

The primary analyses were based on participants with complete data on sleep exposures and covariates. To evaluate the potential influence of missing data, we summarised missingness of key variables and compared included participants with those excluded due to missingness using standardised mean differences. Multiple imputation was additionally performed as a sensitivity analysis, with the outcome, follow-up time, sleep variables, and covariates included in the imputation model; pooled estimates were obtained using Rubin’s rules. We also conducted a series of sensitivity analyses, which were used to test robustness rather than provide independent confirmatory tests.Firstly, participants who developed obesity within the first two years post-enrolment were excluded to mitigate reverse causality bias. Secondly, sleep duration was trimmed at the 1% extreme values. Additionally, the waist-to-hip ratio (WHR) was incorporated as an extra covariate in Model 3 for adjustment. Finally, among participants with repeat measured BMI data, we performed a sensitivity analysis using BMI ≥30 kg/m² at repeat assessment as an alternative obesity outcome. We further repeated the fully adjusted Cox analyses with baseline BMI included as a continuous covariate to examine whether the associations were independent of baseline proximity to the obesity threshold. Because the exact date when BMI first reached 30 kg/m² was unavailable, this analysis was conducted using multivariable logistic regression rather than Cox regression. The same covariate adjustment set as the primary analysis was used.

All statistical analyses were performed using R software (version 4.2.3). The random seed was fixed at 20250611 for multiple imputation and bootstrap-based indirect-association analyses. Key package versions and session information are provided in **Supplementary Table S22** and in the public code repository. Effect estimates were presented with 95% confidence intervals. P values were reported as continuous measures of compatibility with the data rather than as the sole basis for dichotomous interpretation. The study report adheres to the STROBE guidelines (Strengthening the Reporting of Observational Studies in Epidemiology) (Detailed information in **Supplementary Material**, **Supplementary Table S2**).

## 3 Results

### 3.1 Characteristics of the study population

This study included 68, 419 postmenopausal women with a median follow-up of 13.33 years, among whom 1, 146 ICD-coded incident obesity cases were documented. Compared with participants who did not develop ICD-coded obesity, those who developed obesity had slightly shorter mean sleep duration (7.1 ± 1.2 vs. 7.2 ± 1.0 h), higher proportions of usual insomnia (38% vs. 32%), snoring (31% vs. 26%), evening chronotype (38% vs. 33%), and usual daytime sleepiness (2.9% vs. 2.1%). They also had higher mean age, BMI, Townsend Deprivation Index, educational score, and higher proportions of current smoking, diabetes, hypertension, and HRT use. The proportion of current drinkers was lower among participants who developed obesity. Baseline differences are summarised in **Table 1** using standardised mean differences. Missingness of key variables and comparisons between included participants and those excluded due to missingness are shown in **Supplementary Table S16–S18**.

**Table 1:** Baseline characteristics.

Characteristic	Overall (N = 68, 419)	Non-obesity (N = 67, 273)	Incident obesity (N = 1, 146)	SMD
**Age, years**	**59.83 (5.44)**	**59.82 (5.44)**	**60.39 (5.56)**	**0.103**
**Ethnicity**				**0.057**
White	66263 (96.8)	65162 (96.9)	1101 (96.1)	
Mixed	281 (0.4)	274 (0.4)	7 (0.6)	
Asian or Asian British	847 (1.2)	831 (1.2)	16 (1.4)	
Black or Black British	400 (0.6)	389 (0.6)	11 (1.0)	
Chinese	215 (0.3)	212 (0.3)	3 (0.3)	
Other ethnic group	413 (0.6)	405 (0.6)	8 (0.7)	
**Alcohol intake**				**0.139**
Never	3138 (4.6)	3072 (4.6)	66 (5.8)	
Previous	2018 (2.9)	1956 (2.9)	62 (5.4)	
Current	63263 (92.5)	62245 (92.5)	1018 (88.8)	
**Smoking status**				**0.184**
Never	40263 (58.8)	39678 (59.0)	585 (51.0)	
Previous	23085 (33.7)	22655 (33.7)	430 (37.5)	
Current	5071 (7.4)	4940 (7.3)	131 (11.4)	
MET* **score**	**12100.11 (12578.50)**	**12111.77 (12596.27)**	**11415.35 (11471.12)**	**0.058**
**Townsend deprivation index**	**-1.78 (2.76)**	**-1.79 (2.76)**	**-1.24 (3.04)**	**0.189**
**Education score**	**11.43 (13.79)**	**11.35 (13.74)**	**16.34 (15.69)**	**0.339**
**Diabetes mellitus**				**0.145**
No	67040 (98.0)	65946 (98.0)	1094 (95.5)	
Yes	1379 (2.0)	1327 (2.0)	52 (4.5)	
**Hypertension**				**0.311**
No	52515 (76.8)	51794 (77.0)	721 (62.9)	
Yes	15904 (23.2)	15479 (23.0)	425 (37.1)	
**Body mass index, kg/m²**	**24.89 (2.69)**	**24.84 (2.67)**	**27.71 (1.94)**	**1.226**
**Waist-to-hip ratio**	**0.81 (0.06)**	**0.81 (0.06)**	**0.84 (0.07)**	**0.448**
**Healthy diet score**	**3.14 (1.19)**	**3.14 (1.19)**	**3.04 (1.17)**	**0.083**
**Diet quality**				**0.079**
Healthy diet	27824 (40.7)	27401 (40.7)	423 (36.9)	
Intermediate diet	34372 (50.2)	33763 (50.2)	609 (53.1)	
Poor diet	6223 (9.1)	6109 (9.1)	114 (9.9)	
**Number of live births**	**1.85 (1.14)**	**1.85 (1.14)**	**1.92 (1.21)**	**0.056**
**Age at menarche, years**	**12.78 (2.51)**	**12.78 (2.51)**	**12.69 (2.52)**	**0.034**
**Pregnancy loss or termination**				**0.055**
No	47048 (68.8)	46289 (68.8)	759 (66.2)	
Yes	21371 (31.2)	20984 (31.2)	387 (33.8)	
**Oral contraceptive use**				**0.039**
No	13230 (19.3)	12991 (19.3)	239 (20.9)	
Yes	55189 (80.7)	54282 (80.7)	907 (79.1)	
**Hormone replacement therapy use**				**0.149**
No	37954 (55.5)	37402 (55.6)	552 (48.2)	
Yes	30465 (44.5)	29871 (44.4)	594 (51.8)	
**Sleep duration, h/day**	**7.19 (1.04)**	**7.19 (1.04)**	**7.12 (1.20)**	**0.068**
**Sleep duration category**				**0.143**
7–9 h/day	51808 (75.7)	51006 (75.8)	802 (70.0)	
≤6 h/day	15664 (22.9)	15349 (22.8)	315 (27.5)	
≥10 h/day	947 (1.4)	918 (1.4)	29 (2.5)	
**Insomnia symptoms**				**0.147**
Never/rarely	11399 (16.7)	11241 (16.7)	158 (13.8)	
Sometimes	35243 (51.5)	34696 (51.6)	547 (47.7)	
Usually	21777 (31.8)	21336 (31.7)	441 (38.5)	
**Chronotype**				**0.109**
Morning/intermediate chronotype	45610 (66.7)	44905 (66.8)	705 (61.5)	
Evening chronotype	22809 (33.3)	22368 (33.2)	441 (38.5)	
**Snoring**				**0.130**
No	50754 (74.2)	49970 (74.3)	784 (68.4)	
Yes	17665 (25.8)	17303 (25.7)	362 (31.6)	
**Daytime sleepiness**				**0.076**
Never/rarely	53367 (78.0)	52504 (78.0)	863 (75.3)	
Sometimes	13622 (19.9)	13373 (19.9)	249 (21.7)	
Often/all of the time	1430 (2.1)	1396 (2.1)	34 (3.0)	

*MET, Metabolic Equivalent of Task.

Bold font indicates statistical significance at the p < 0.05 level.

### 3.2 Association between different sleep behaviours and obesity in postmenopausal women

In the fully adjusted Cox model (**Table 2**), both short sleep duration (≤6 h; HR 1.214, 95% CI 1.065-1.384) and long sleep duration (≥10 h; HR 1.654, 95% CI 1.141-2.399) were associated with a higher rate of ICD-coded incident obesity compared with 7–9 h of sleep. Positive associations were also observed for usual insomnia symptoms (HR 1.320, 95% CI 1.100-1.585), evening chronotype (HR 1.224, 95% CI 1.085-1.380), and snoring (HR 1.234, 95% CI 1.089-1.399). Estimates for occasional insomnia and daytime sleepiness were less precise, with confidence intervals compatible with weak associations or no clear association after full adjustment. Sensitivity analyses excluding obesity cases occurring within the first 2 years, trimming extreme sleep-duration values, and additionally adjusting for waist-to-hip ratio yielded broadly similar results (**Supplementary Tables S3–S5**). In the subset with repeat BMI measurements, logistic regression using BMI ≥30 kg/m² at repeat assessment as an alternative outcome showed partially consistent results, with similar positive associations for short sleep and usual insomnia but attenuated or inconsistent estimates for other sleep behaviours (**Supplementary Table S19**). Sensitivity analyses are presented in **Supplementary Tables S3–S5** and **S18–S19**. Additional adjustment for baseline BMI as a continuous covariate is shown in **Supplementary Table S23**. Proportional hazards diagnostics based on Schoenfeld residuals are summarised in **Supplementary Table S21**. Mild departures were observed for snoring and some global tests, but the main sleep-exposure estimates were retained and interpreted with caution. Some subgroup estimates, particularly those for long sleep duration, were based on small numbers of events and had wide confidence intervals. These estimates were therefore not emphasised as substantive findings.

**Table 2:** Associations between sleep behaviours and incident obesity in postmenopausal women.

Characteristic	Model 1 HR (95% CI); *p*	Model 2 HR (95% CI); *p*	Model 3 HR (95% CI); *p*
Group of sleep durations			
7–9 hours	Ref.	Ref.	Ref.
≤6h	1.298(1.139~1.479); <0.001	1.220(1.070~1.390); 0.003	**1.214(1.065~1.384); 0.004**
≥10 hours	1.948(1.345~2.823); < 0.001	1.652(1.139~2.396); 0.008	**1.654(1.141~2.399);** **0.008**
Insomnia			
Never/rarely	Ref.	Ref.	Ref.
Occasionally	1.115(0.934~1.331); 0.23	1.077(0.902~1.286); 0.413	1.069(0.895~1.276); 0.463
Usually	1.473(1.228~1.767); <0.001	1.344(1.120~1.613); 0.001	**1.320(1.100~1.585); 0.003**
Chronotype			
Morning	Ref.	Ref.	Ref.
Evening	1.265(1.123~1.425); <0.001	1.225(1.086~1.381); 0.001	**1.224(1.085~1.380); 0.001**
Snoring			
No	Ref.	Ref.	Ref.
Yes	1.331(1.175~1.508); <0.001	1.238(1.092~1.403); 0.001	**1.234(1.089~1.399);** **0.001**
Daytime drowsiness, N (%)			
Never/rarely	Ref.	Ref.	Ref.
Occasionally	1.100(0.954~1.267); 0.188	1.038(0.900~1.196); 0.61	1.035(0.898~1.194); 0.633
Usually	1.454(1.032~2.049); 0.032	1.271(0.901~1.793); 0.171	1.260(0.894~1.778); 0.187

*Notes: Model 1 was adjusted for age and ethnicity. Model 2 was additionally adjusted for educational score, Townsend Deprivation Index, smoking status, alcohol drinking status, MET score, healthy diet score, diabetes, and hypertension. Model 3 was further adjusted for number of live births, age at menarche, pregnancy-loss history, oral contraceptive use, and HRT use. Sleep duration was modelled categorically in **Table 2**; the nonlinear association with continuous sleep duration was assessed using restricted cubic splines in **Figure 1**. P values are reported as continuous measures of compatibility with the data. Abbreviations: BMI, body mass index; CI, confidence interval; HR, hazard ratio; HRT, hormone replacement therapy; MET, metabolic equivalent of task; TDI, Townsend Deprivation Index.

Bold font indicates statistical significance at the p < 0.05 level.

**Figure 1:**
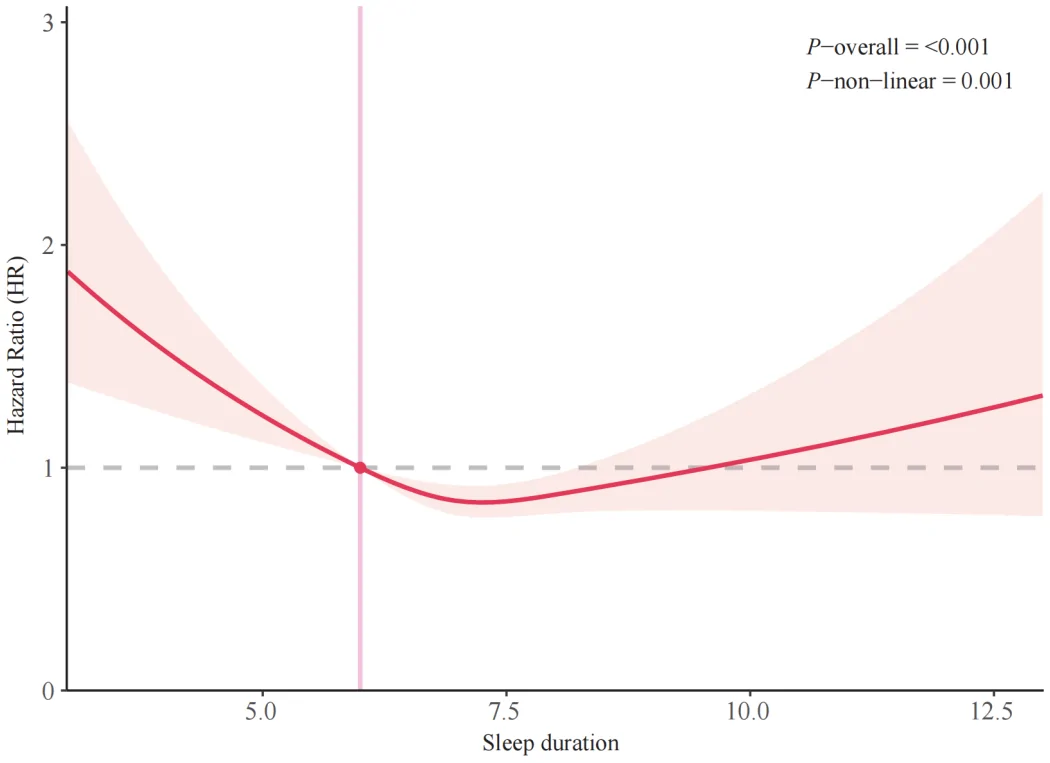
Restricted cubic spline analysis of sleep duration and incident obesity in postmenopausal women. The red solid line in the figure represents the estimated hazard ratio (HR) curve, and the shaded area denotes the corresponding 95% confidence interval.

Stratified estimates are shown in **Supplementary Tables S6–S12**. These analyses were interpreted descriptively rather than by comparing whether p values crossed a fixed threshold across subgroups. Formal interaction tests are presented in **Supplementary Table S20**. Most interaction tests did not suggest clear heterogeneity; however, exploratory signals were observed for chronotype across HRT use, hypertension history, and TDI tertile, and for snoring across ethnicity. Given the exploratory nature of these tests and the imprecision of several subgroup estimates, particularly for long sleep duration and smaller strata, these findings were interpreted cautiously and were not considered confirmatory evidence of effect modification. Restricted cubic spline analysis showed an approximately U-shaped nonlinear association between sleep duration and ICD-coded incident obesity (P-overall < 0.001; P-nonlinearity = 0.001), with the lowest estimated risk around 7 hours of sleep (**Figure 1**).

### 3.3 Exploratory indirect-association analysis

**Figure 2:**
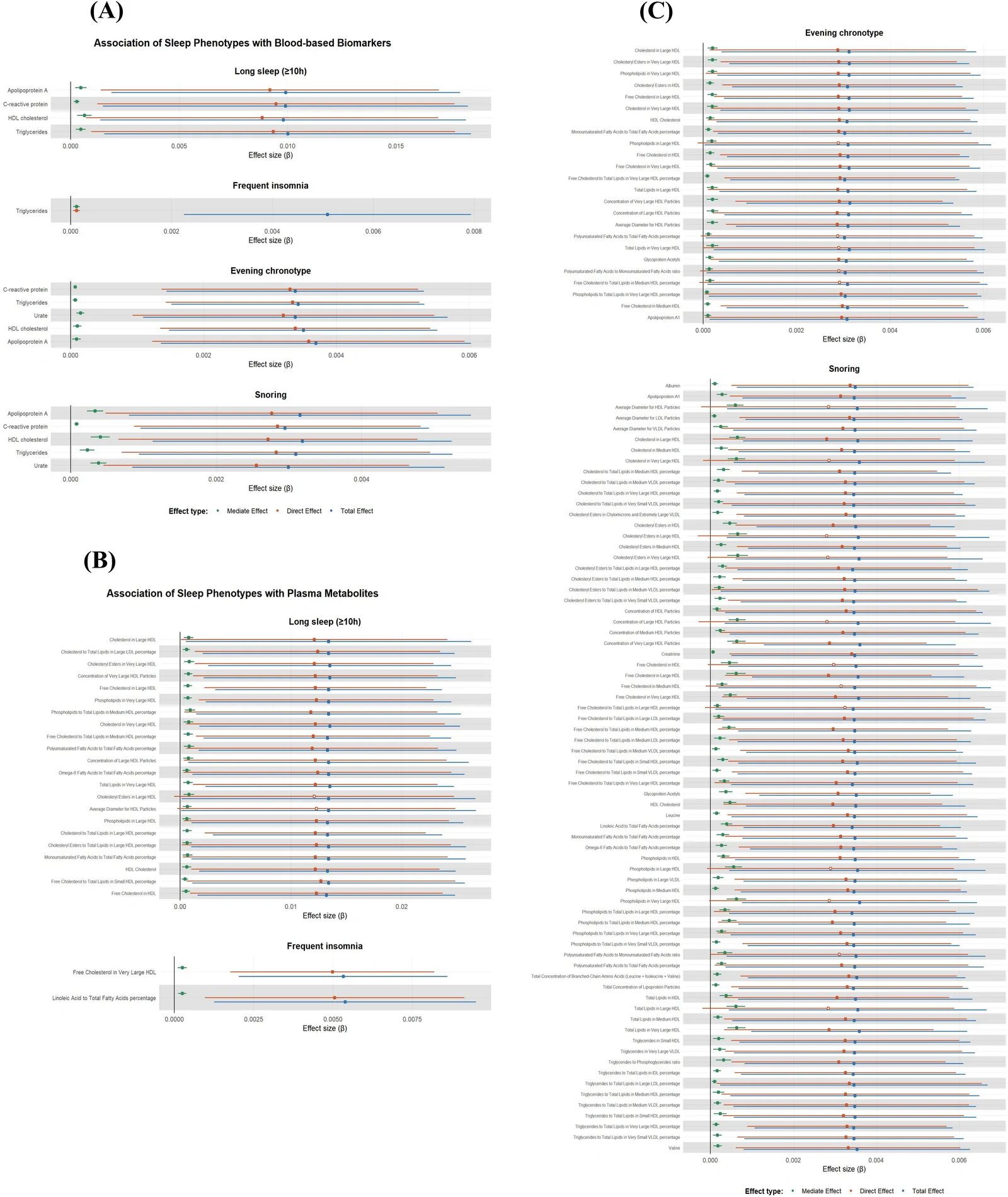
Exploratory indirect-association analyses of biomarkers and metabolites in the sleep–obesity association. *Note: Exploratory indirect-association analyses of selected biomarkers and metabolites in the sleep–obesity association. The figure displays selected nominal indirect-association signals retained for visualisation based on uncorrected indirect-effect p values. Complete indirect-association results, including FDR-adjusted p values, are provided in **Supplementary Tables S13–S14**. These analyses were exploratory and should not be interpreted as evidence of causal mediation.

We selected blood biomarkers (white blood cell count, neutrophil count, lymphocyte count, monocyte count, platelet count, red cell distribution width (RDW), mean platelet volume (MPV), plateletcrit (PCT), platelet distribution width (PDW), C-reactive protein (CRP), triglycerides, total cholesterol, low-density lipoprotein (LDL), high-density lipoprotein (HDL), apolipoprotein A, apolipoprotein B, lipoprotein A, glycated haemoglobin, fasting blood glucose, and uric acid) and 249 plasma metabolites as potential mediating variables to construct mediation analysis models (**Supplementary Materials**; **Supplementary Table S13-14**). (Results are presented in **Figure 2**.) Exploratory indirect-association analyses are summarised in **Figure 2** and **Supplementary Table S13–S14**. Overall, the estimated indirect effects were small relative to the total associations. Nominal signals were mainly observed for inflammation- and lipid-related biomarkers and metabolites. However, most findings were attenuated after FDR correction; therefore, these results should be interpreted as hypothesis-generating biological clues rather than evidence of causal mediation or identified biological pathways. Actual sample sizes and event counts for the plasma metabolomic indirect-association analyses are reported in **Supplementary Table S24**.

## 4 Discussion

Based on the UK Biobank large prospective cohort, this study systematically evaluated the relationship between multidimensional sleep behaviour and new-onset obesity in 68, 419 postmenopausal women with baseline non-obesity, and further used blood biomarkers and plasma metabolites for exploratory indirect-association analyses. We observed an approximately U-shaped association between sleep duration and incident obesity: compared with 7–9 hours of sleep per night, both short sleep (≤ 6 hours) and long sleep (≥ 10 hours) were associated with increased obesity incidence. This finding aligns with reports from broader populations (7, 8). More significantly, we observed that poor sleep quality and circadian rhythm disruption are equally independent and potent risk factors: frequent insomnia, nocturnal rhythm preference, and snoring were associated with approximately 32%, 22%, and 23% increased obesity risk, respectively. These findings underscore that obesity risk in postmenopausal women is influenced not only by sleep duration but also by sleep quality and rhythmic characteristics. Consequently, we further investigated potential biological correlates of the associations between different sleep phenotypes and ICD-coded incident obesity. Our results revealed that CRP, triglycerides, HDL-C, apo A, HDL-related metabolites, and unsaturated fatty acid ratios showed nominal indirect associations with these sleep–obesity associations. The overall findings suggest that obesity risk in postmenopausal women is closely associated with sleep duration, quality, and rhythmic characteristics.

Exploratory biomarker and metabolomic analyses provided limited biological clues suggesting possible involvement of inflammation and lipid metabolism, although the indirect effects were small.The biomarker and metabolomic analyses should be interpreted cautiously. Because sleep behaviours and candidate intermediate variables were measured at the same baseline assessment, these analyses cannot establish that biomarkers or metabolites lie downstream of sleep behaviours. Reverse directionality and residual confounding remain possible. In addition, the indirect-effect estimates were obtained on an additive probability-difference scale and were generally small; most did not remain robust after FDR correction. Therefore, these findings provide only limited, hypothesis-generating biological clues related to inflammation and lipid metabolism, rather than evidence of causal mediation.

Previous literature suggests that sleep disorders may influence weight regulation through multiple mechanisms, including alterations in neuroendocrine function, disruption of energy balance, and activation of reward systems. On the one hand, dysregulation of appetite hormones (such as decreased leptin levels and elevated ghrelin) may activate the hypothalamic orexin system, thereby promoting feeding behaviour, prolonging wakefulness, and reducing energy expenditure (9–12). Conversely, circadian rhythm disruption impairs hypothalamic-pituitary-adrenal (HPA) axis and hypothalamic-pituitary-thyroid (HPT) axis function, triggering downstream hormonal dysregulation that further increases fat synthesis (13). Moreover, sleep abnormalities may enhance the rewarding effects of food by activating brain regions such as the striatum and amygdala, thereby promoting excessive food intake and increasing preference for high-calorie foods (14). Concurrently, individuals with sleep disorders are more prone to comorbid metabolic or psychiatric conditions (such as depression, obstructive sleep apnoea syndrome, or hypothyroidism), which themselves are closely associated with obesity (15–18). It is noteworthy that the aforementioned mechanisms are likely significantly amplified in the postmenopausal population of this study. Menopause-related endocrine changes are associated with increases in total and regional adiposity and a shift toward central fat accumulation (19–21). Vasomotor symptoms and other menopause-related complaints are also closely linked to sleep disturbance, which may further worsen metabolic health in this population (22). Although this study could not directly verify all the above paths, the strong correlation between multidimensional sleep abnormalities and obesity risk was observed in postmenopausal women, which was consistent with the hypothesis that the “susceptible background is magnified”.

In exploratory indirect-association analyses, several inflammation- and lipid-related biomarkers showed nominal indirect associations with sleep phenotypes and incident obesity. At the level of conventional blood biomarkers, we observed that C-reactive protein (CRP), triglycerides (TG), high-density lipoprotein cholesterol (HDL-C), and apolipoprotein A (apo A) showed nominal indirect signals in the associations between long sleep duration, night-type rhythm, and snoring with obesity. Triglycerides also showed nominal indirect signals for the association between frequent insomnia and obesity, suggesting systemic low-grade inflammation and lipid metabolism disorders may be consistent with possible involvement of low-grade inflammation and lipid metabolism in the sleep–obesity association. At the plasma metabolite level, we observed nominal indirect-association signals involving HDL-associated metabolites and mono/polyunsaturated fatty acid ratios. Integrating prior research, intermittent hypoxia, oxidative stress, and chronic inflammation induced by sleep disorders (including OSA) (23, 24). significantly suppress hepatic apolipoprotein A-I (apoA-I) expression, and impair HDL’s cholesterol reverse transport capacity and antioxidant function (25, 26). Concurrently, the decline in oestrogen levels during menopause further reduces HDL levels and the proportion of large-particle HDL2 subtypes (27, 28). These alterations may collectively diminish HDL’s protective effects on lipid metabolism and anti-inflammation, thereby increasing the risk of obesity and metabolic abnormalities (29). In addition, this study observed nominal indirect signals for branched-chain amino acids in the snoring-obesity pathway, which also echoes the existing reports on the role of branched-chain amino acids (BCAAs)in mTORC1 activation and insulin resistance (30–35). In summary, from conventional biochemical indicators to plasma metabolites, these exploratory findings are broadly consistent with the hypothesis that sleep disrupts metabolic health through multiple pathways, including inflammation, lipid metabolism, and amino acid metabolism. These findings are exploratory and should not be interpreted as proof of biological mechanisms or causal mediation. Although several biomarkers and metabolites showed nominal indirect associations, the estimated mediation proportions were small and most metabolite findings did not remain robust after FDR correction. Therefore, these results should be interpreted as exploratory biological clues rather than evidence of causal mediation. Other unmeasured pathways, residual confounding, and reverse directionality remain possible.Stratified analyses were exploratory. Although some estimates appeared to vary across subgroups, formal interaction tests did not provide strong evidence of material effect modification. Therefore, these findings should be interpreted as descriptive and hypothesis-generating rather than evidence that age, baseline BMI, HRT use, ethnicity, diabetes, hypertension, or socioeconomic deprivation modified the sleep–obesity association.

This study is based on the UK Biobank large prospective cohort, with a large sample size and long follow-up time, which can stably evaluate the time association between sleep behaviour and new-onset obesity. Secondly, this study depicts sleep characteristics from multiple dimensions, such as sleep duration, insomnia, circadian rhythm types, snoring, and daytime sleepiness, which is more comprehensive than previous studies that only focused on a single indicator. Third, for the first time, this study combined blood biomarkers and metabolomics data to conduct mediation analysis in a large-scale postmenopausal female population, providing systematic evidence for understanding the metabolic pathways behind the sleep-obesity relationship. Several limitations should be noted. First, the primary outcome was ICD-coded incident obesity based on hospital admission and death-registry records. This definition is likely to have high specificity but limited sensitivity, because some participants who reached BMI ≥30 kg/m² may not have received an obesity diagnosis code. Therefore, our outcome should be interpreted as clinically recorded obesity rather than all BMI-defined incident obesity. Differential ascertainment is also possible, as participants with higher baseline BMI or comorbidities may have more clinical contact and thus a higher probability of receiving an obesity code. Although we performed a sensitivity analysis using repeat measured BMI ≥30 kg/m², repeat BMI was available only in a subset, and these results should also be interpreted cautiously. The primary analysis used a complete-case approach. Although we compared included and excluded participants and performed multiple-imputation sensitivity analyses, selection bias due to missingness cannot be fully excluded. Age at menopause and years since menopause were not included in the models; therefore, residual confounding related to menopausal timing cannot be excluded. The fully adjusted models should not be interpreted as estimating a definitive causal total effect. Some adjusted variables, such as diabetes and hypertension, may partly lie on pathways related to sleep and obesity, and adjustment for them may attenuate the total association. The exploratory indirect-association analyses also require caution. Causal mediation would require strong assumptions, including no unmeasured exposure–mediator, mediator–outcome, or exposure–outcome confounding, which cannot be verified in this observational study.

Nevertheless, this study offers clinical implications for obesity prevention and sleep health management in postmenopausal women. Firstly, the findings support systematically incorporating sleep behaviour assessments into obesity and metabolic risk evaluations for this population, with particular attention to phenotypes such as short or long sleep duration, frequent insomnia, nocturnal rhythm preference, and snoring. Individuals exhibiting potential sleep-disordered breathing should undergo further screening. Secondly, from an intervention perspective, incorporating regular sleep schedule guidance, sleep hygiene education, and comprehensive management of menopausal symptoms into weight management programmes for postmenopausal women may effectively reduce obesity risk in this population.

## 5 Conclusions

Abnormal sleep duration, usual insomnia, evening chronotype, and snoring were associated with a higher rate of incident obesity in postmenopausal women. Exploratory biomarker and metabolomic analyses provided limited, hypothesis-generating clues related to inflammation and lipid metabolism. These findings support the potential value of incorporating sleep health assessment into obesity prevention strategies for postmenopausal women.

## Data Availability

The datasets presented in this study can be found in online repositories. The names of the repository/repositories and accession number(s) can be found in the article/**Supplementary Material**.
