# Supplementary material for: Multidimensional sleep behaviours and incident obesity in postmenopausal women: a prospective cohort study
Source: Front Endocrinol (Lausanne). 2026 Aug 17;17:1890100. doi: 10.3389/fendo.2026.1890100 (PMC13526600; doi:10.3389/fendo.2026.1890100)
Supplement: Supplementary file 1 [file Supplementaryfile1.docx]

# **Multidimensional Sleep Behaviors and Incident Obesity in Postmenopausal Women: A Prospective Cohort Study**

**Supplementary material**

**Supplementary content**

Figure S1. Flowchart for inclusion and exclusion of participants

Table S1. Covariates definitions used in the UK Biobank study

Table S2. STROBE Checklist

Table S3. Sensitivity analysis after excluding participants who developed obesity within the first 2 years of follow-up

Table S4 .Sensitivity analysis after trimming the top and bottom 1% of sleep duration

Table S5.Sensitivity analysis with further adjustment for waist-to-hip ratio (WHR) in Model 3

Table S6. Stratified Analysis by Age Group

Table S7. Stratified Analysis by Baseline BMI Category

Table S8. Stratified Analysis by Hormone Replacement Therapy (HRT) Use

Table S9. Stratified Analysis by Ethnicity

Table S10. Stratified by Diabetes History

Table S11. Stratified by Hypertension History

Table S12. Stratified Analysis by Townsend Deprivation Index (TDI) Tertile

#### **Figure S1. Flowchart for inclusion and exclusion of participants**

#### **Table S1. Covariates definitions used in the UK Biobank study**

| **Covariate** | **UK Biobank variable**  **name** | **Detailed description** |
| --- | --- | --- |
| Age | 21022 | Continuous variable  Dummy variable:  0: <60 years  1: ≥60 years |
| Ethnicity | 21000 | Dummy variable:  0: White  1: Mixed  2: Asian or Asian British  3: Black or Black British  4: Chinese  5: Other ethnic group |
| Educational Score | 26414,  26421,  26431 | Continuous variable |
| TDI | 189 | Continuous variable |
| Smoking status | 20116 | Dummy variable:  0: never  1: previous  2: current |
| Drinking status | 20117 | Dummy variable:  0: never  1: previous  2: current |
| MET score | 22037,  22038,  22039 | Continuous variable |
| Diet | 1289, 1299,1309, 1329, 1339, 1349, 1359, 1369, 1379, 1389 | Data derived from the UK Biobank food-frequency questionnaire were used to allocate one point for each of the following healthy dietary components. Individual component scores were then summed to produce a healthy diet score ranging from 0 to 5, with higher scores indicating a healthier overall diet. Participants were subsequently classified into three diet-quality categories based on their healthy diet score: healthy diet (score ≥ 4), intermediate diet (score 2–3) and poor diet (score ≤ 1).  Scoring criteria (one point awarded for each criterion met; total score = sum of individual points):  Fruits: ≥ 3 servings/day  Vegetables: > 3 servings/day  Fish: ≥ 2 servings/week  Processed meats: ≤ 1 serving/week  Unprocessed red meats: ≤ 1.5 servings/week  Dummy variable coding:  0: Healthy diet  1: Intermediate diet  2: Poor diet |
| Diabetes | 6153, 6177  ICD10-code: E10, E11, E12, E13, E14 | Dummy variable:  0: No  1: Yes |
| Hypertension | 6150, 6153, 6177  ICD10-code: I10 | Dummy variable:  0: No  1: Yes |
| Menopause | 2724 | Dummy variable:  0: No  1: Yes  2: Not sure |
| Number of live births | 2734 | Continuous variable |
| Age at menarche | 2714 | Continuous variable |
| Stillbirth/Spontaneous miscarriage/Termination | 2774 | Dummy variable:  0: No  1: Yes |
| Oral contraceptive | 2784 | Dummy variable:  0: No  1: Yes |
| HRT | 2814 | Dummy variable:  0: No  1: Yes |
| **Note: The educational score is derived from the educational variables in the UK Biobank; a higher score indicates a higher level of education. This variable is analyzed as a continuous covariate* | | |

#### **Table S2****. STROBE Checklist.**

|  | **Item No** | **Recommendation** | **Section/ Exhibit** |
| --- | --- | --- | --- |
| **Title and abstract** | 1 | (*a*) Indicate the study’s design with a commonly used term in the title or the abstract | Title |
|  |  | (*b*) Provide in the abstract an informative and balanced summary of what was done and what was found | Abstract |
| **Introduction** | | |  |
| Background/rationale | 2 | Explain the scientific background and rationale for the investigation being reported | Introduction |
| Objectives | 3 | State specific objectives, including any prespecified hypotheses | Introduction |
| **Methods** | | |  |
| Study design | 4 | Present key elements of study design early in the paper | Methods 2.1，Figure S1 |
| Setting | 5 | Describe the setting, locations, and relevant dates, including periods of recruitment, exposure, follow-up, and data collection | Methods 2.1-2.4 |
| Participants | 6 | (*a*) Give the eligibility criteria, and the sources and methods of selection of participants. Describe methods of follow-up | Methods 2.1,Figure S1 |
|  |  | (*b*) For matched studies, give matching criteria and number of exposed and unexposed | Not applicable |
| Variables | 7 | Clearly define all outcomes, exposures, predictors, potential confounders, and effect modifiers. Give diagnostic criteria, if applicable | Methods 2.2-2.4, Supplementary Table S1 |
| Data sources/ measurement | 8* | For each variable of interest, give sources of data and details of methods of assessment (measurement). Describe comparability of assessment methods if there is more than one group | Methods 2.2,2.4 |
| Bias | 9 | Describe any efforts to address potential sources of bias | Methods 2.5 |
| Study size | 10 | Explain how the study size was arrived at | Methods 2.1, Results 3.1 |
| Quantitative variables | 11 | Explain how quantitative variables were handled in the analyses. If applicable, describe which groupings were chosen and why | Methods 2.2, Supplementary Table S1 |
| Statistical methods | 12 | (*a*) Describe all statistical methods, including those used to control for confounding | Methods 2.5 |
|  |  | (*b*) Describe any methods used to examine subgroups and interactions | Methods 2.5 |
|  |  | (*c*) Explain how missing data were addressed | Methods 2.1 and 2.5；Supplementary Tables S16–S18 |
|  |  | (*d*) If applicable, explain how loss to follow-up was addressed | Not applicable |
|  |  | (*e*) Describe any sensitivity analyses | Methods 2.5 |
| **Results** | | |  |
| Participants | 13* | (a) Report numbers of individuals at each stage of study—eg numbers potentially eligible, examined for eligibility, confirmed eligible, included in the study, completing follow-up, and analysed | Figure S1 |
|  |  | (b) Give reasons for non-participation at each stage | Figure S1 |
|  |  | (c) Consider use of a flow diagram | Figure S1 |
| Descriptive data | 14* | (a) Give characteristics of study participants (eg demographic, clinical, social) and information on exposures and potential confounders | Results 3.1, Table 1 |
|  |  | (b) Indicate number of participants with missing data for each variable of interest | Figure S1, Supplementary Tables S16-S18 |
|  |  | (c) Summarise follow-up time (eg, average and total amount) | Results 3.1 |
| Outcome data | 15* | Report numbers of outcome events or summary measures over time | Result 3.1 |
| Main results | 16 | (*a*) Give unadjusted estimates and, if applicable, confounder-adjusted estimates and their precision (eg, 95% confidence interval). Make clear which confounders were adjusted for and why they were included | Result 3.2,Table 2 |
|  |  | (*b*) Report category boundaries when continuous variables were categorized | Methods2.2 |
|  |  | (*c*) If relevant, consider translating estimates of relative risk into absolute risk for a meaningful time period | Not applicable |
| Other analyses | 17 | Report other analyses done—eg analyses of subgroups and interactions, and sensitivity analyses | Results 3.2-3.3.Supplementary Tables S3-S12, S19, S20 |
| **Discussion** | | |  |
| Key results | 18 | Summarise key results with reference to study objectives | Discussion |
| Limitations | 19 | Discuss limitations of the study, taking into account sources of potential bias or imprecision. Discuss both direction and magnitude of any potential bias | Discussion |
| Interpretation | 20 | Give a cautious overall interpretation of results considering objectives, limitations, multiplicity of analyses, results from similar studies, and other relevant evidence | Discussion,  Conclusion |
| Generalisability | 21 | Discuss the generalisability (external validity) of the study results | Discussion |
| **Other information** | | |  |
| Funding | 22 | Give the source of funding and the role of the funders for the present study and, if applicable, for the original study on which the present article is based | **Funding statement** |
| **Give information separately for exposed and unexposed groups. Note: An Explanation and Elaboration article discusses each checklist item and gives methodological background and published examples of transparent reporting. The STROBE checklist is best used in conjunction with this article (freely available on the Web sites of PLoS Medicine at http://www.plosmedicine.org/, Annals of Internal Medicine at http://www.annals.org/, and Epidemiology at http://www.epidem.com/). Information on the STROBE Initiative is available at http://www.strobe-statement.org.* | | | |

**Table S3. Sensitivity analysis after excluding participants who developed obesity within the first 2 years of follow-up**

|  | **Model 1** | **Model 2** | **Model 3** |
| --- | --- | --- | --- |
|  | **HR (95%CI); *p*** | **HR (95%CI); *p*** | **HR (95%CI); *p*** |
| **Group of sleep durations** |  |  |  |
| 7-9h | Ref. | Ref. | Ref. |
| ≤6h | 1.301(1.140~1.485);<0.001 | 1.222(1.071~1.396);0.003 | **1.218(1.066~1.390);0.004** |
| ≥10h | 1.932(1.325~2.817);0.001 | 1.647(1.128~2.403);0.01 | **1.647(1.128~2.404);0.01** |
| **Insomnia** |  |  |  |
| Never/rarely | Ref. | Ref. | Ref. |
| Occasionally | 1.124(0.939~1.345);0.204 | 1.084(0.906~1.298);0.379 | 1.076(0.898~1.288);0.428 |
| Usually | 1.477(1.228~1.776);<0.001 | 1.346(1.118~1.620);0.002 | **1.321(1.097~1.591);0.003** |
| **Chronotype** |  |  |  |
| Morning | Ref. | Ref. | Ref. |
| Evening | 1.271(1.127~1.434);<0.001 | 1.234(1.092~1.394);0.001 | **1.233(1.091~1.393);0.001** |
| **Snoring** |  |  |  |
| No | Ref. | Ref. | Ref. |
| Yes | 1.322(1.165~1.500);<0.001 | 1.230(1.084~1.397);0.001 | **1.226(1.080~1.393);0.002** |
| **Daytime dozing** |  |  |  |
| Never/rarely | Ref. | Ref. | Ref. |
| Occasionally | 1.098(0.951~1.268);0.202 | 1.037(0.898~1.198);0.617 | 1.036(0.897~1.197);0.631 |
| Usually | 1.498(1.063~2.111);0.021 | 1.316(0.933~1.856);0.118 | 1.305(0.925~1.841);0.129 |
| **Notes: HR=hazard ratio; CI=confidence interval.*  *Model 1 was adjusted for age and ethnicity. Model 2 was additionally adjusted for educational score, Townsend Deprivation Index, smoking status, alcohol drinking status, MET score, healthy diet score, diabetes, and hypertension. Model 3 was further adjusted for number of live births, age at menarche, pregnancy-loss history, oral contraceptive use, and HRT use.* | | | |

**Table S4. Sensitivity analysis after trimming the top and bottom 1% of sleep duration**

|  | **Model 1** | **Model 2** | **Model 3** |
| --- | --- | --- | --- |
|  | **HR (95%CI); *p*** | **HR (95%CI); *p*** | **HR (95%CI); *p*** |
| **Group of sleep durations** |  |  |  |
| 7-9h | Ref. | Ref. | Ref. |
| ≤6h | 1.271(1.112~1.452);<0.001 | 1.202(1.052~1.374);0.007 | **1.198(1.048~1.369);0.008** |
| ≥10h | 1.866(1.233~2.826);0.003 | 1.608(1.061~2.436);0.025 | **1.613(1.065~2.444);0.024** |
| **Insomnia** |  |  |  |
| Never/rarely | Ref. | Ref. | Ref. |
| Occasionally | 1.118(0.936~1.337);0.218 | 1.079(0.903~1.290);0.401 | 1.071(0.896~1.280);0.452 |
| Usually | 1.469(1.223~1.765);<0.001 | 1.344(1.118~1.616);0.002 | **1.320(1.097~1.587);0.003** |
| **Chronotype** |  |  |  |
| Morning | Ref. | Ref. | Ref. |
| Evening | 1.254(1.112~1.415);<0.001 | 1.211(1.072~1.368);0.002 | **1.211(1.072~1.368);0.002** |
| **Snoring** |  |  |  |
| No | Ref. | Ref. | **Ref.** |
| Yes | 1.324(1.167~1.501);<0.001 | 1.229(1.083~1.395);0.001 | **1.225(1.079~1.391);0.002** |
| **Daytime dozing** |  |  |  |
| Never/rarely | Ref. | Ref. | Ref. |
| Occasionally | 1.094(0.948~1.263);0.219 | 1.032(0.894~1.192);0.665 | 1.030(0.892~1.189);0.691 |
| Usually | 1.422(0.999~2.024);0.051 | 1.254(0.880~1.786);0.211 | 1.241(0.871~1.768);0.233 |
| **Notes: HR=hazard ratio; CI=confidence interval.*  *Model 1 was adjusted for age and ethnicity. Model 2 was additionally adjusted for educational score, Townsend Deprivation Index, smoking status, alcohol drinking status, MET score, healthy diet score, diabetes, and hypertension. Model 3 was further adjusted for number of live births, age at menarche, pregnancy-loss history, oral contraceptive use, and HRT use.* | | | |

**Table S5. Sensitivity analysis with further adjustment for waist-to-hip ratio (WHR) in Model 3**

|  | **Model 1** | **Model 2** | **Model 3** |
| --- | --- | --- | --- |
|  | **HR (95%CI); *p*** | **HR (95%CI); *p*** | **HR (95%CI); *p*** |
| **Group of sleep durations** |  |  |  |
| 7-9h | Ref. | Ref. | Ref. |
| ≤6h | 1.298(1.139~1.479);<0.001 | 1.220(1.070~1.390);0.003 | **1.209(1.060~1.378);0.005** |
| ≥10h | 1.948(1.345~2.823);<0.001 | 1.652(1.139~2.396);0.008 | **1.560(1.075~2.262);0.019** |
| **Insomnia** |  |  |  |
| Never/rarely | Ref. | Ref. | Ref. |
| Occasionally | 1.115(0.934~1.331);0.23 | 1.077(0.902~1.286);0.413 | 1.076(0.901~1.285);0.42 |
| Usually | 1.473(1.228~1.767);<0.001 | 1.344(1.120~1.613);0.001 | **1.307(1.088~1.569);0.004** |
| **Chronotype** |  |  |  |
| Morning | Ref. | Ref. | Ref. |
| Evening | 1.265(1.123~1.425);<0.001 | 1.225(1.086~1.381);0.001 | **1.207(1.070~1.361);0.002** |
| **Snoring** |  |  |  |
| No | Ref. | Ref. | Ref. |
| Yes | 1.331(1.175~1.508);<0.001 | 1.238(1.092~1.403);0.001 | **1.162(1.025~1.318);0.019** |
| **Daytime dozing** |  |  |  |
| Never/rarely | Ref. | Ref. | Ref. |
| Occasionally | 1.100(0.954~1.267);0.188 | 1.038(0.900~1.196);0.61 | 1.006(0.872~1.160);0.938 |
| Usually | 1.454(1.032~2.049);0.032 | 1.271(0.901~1.793);0.171 | 1.222(0.866~1.723);0.254 |
| **Notes: HR=hazard ratio; CI=confidence interval.*  *Model 1 was adjusted for age and ethnicity. Model 2 was additionally adjusted for educational score, Townsend Deprivation Index, smoking status, alcohol drinking status, MET score, healthy diet score, diabetes, and hypertension. Model 3 was further adjusted for number of live births, age at menarche, pregnancy-loss history, oral contraceptive use, and HRT use.* | | | |

#### **Table S6. Stratified Analysis by Age Group**

| **Characteristic** | **Age (<60 years)** | **Age (≥60 years)** |
| --- | --- | --- |
|  | **HR（95%CI）；*p*** | **HR（95%CI）；*p*** |
| **Group of sleep durations** |  |  |
| 7-9h | Ref. | Ref. |
| ≤6h | **1.388(1.134~1.697);0.001** | 1.106(0.930~1.316);0.254 |
| ≥10h | **1.934(1.081~3.460);0.026** | 1.490(0.918~2.418);0.107 |
| **Insomnia** |  |  |
| Never/rarely | Ref. | Ref. |
| Occasionally | 1.207(0.899~1.621);0.211 | 0.992(0.794~1.239);0.942 |
| Usually | **1.593(1.183~2.145);0.002** | 1.169(0.927~1.476);0.188 |
| **Chronotype** |  |  |
| Morning | Ref. | Ref. |
| Evening | **1.338(1.108~1.617);0.003** | 1.152(0.985~1.348);0.077 |
| **Snoring** |  |  |
| No | Ref. | Ref. |
| Yes | **1.240(1.015~1.513);0.035** | **1.235(1.051~1.452);0.01** |
| **Daytime dozing** |  |  |
| Never/rarely | Ref. | Ref. |
| Occasionally | 1.089(0.857~1.383);0.487 | 1.005(0.842~1.199);0.96 |
| Usually | 1.280(0.747~2.191);0.369 | 1.233(0.787~1.929);0.36 |
| **Note: All models use the fully adjusted Model 3 (adjusted for age, ethnicity, Townsend deprivation index, education, smoking, alcohol, physical activity, diet, medical history, reproductive factors, oral contraceptives, and HRT). p < 0.05 indicates statistically significant results.*  *Abbreviations: HR: Hazard Ratio; CI: Confidence Interval* | | |

**Table S7. Stratified Analysis by Baseline BMI Category**

| **Characteristic** | **BMI (18.5-24.9kg/m^2^)** | **BMI (25.0-29.9kg/m^2^)** |
| --- | --- | --- |
|  | **HR（95%CI）；*p*** | **HR（95%CI）；*p*** |
| **Group of sleep durations** |  |  |
| 7-9h | Ref. | Ref. |
| ≤6h | 1.050(0.697~1.582);0.816 | **1.233(1.073~1.415);0.003** |
| ≥10h | 1.900(0.597~6.044);0.277 | 1.471(0.993~2.177);0.054 |
| **Insomnia** |  |  |
| Never/rarely | Ref. | Ref. |
| Occasionally | 0.918(0.551~1.527);0.741 | 1.100(0.910~1.330);0.322 |
| Usually | 1.135(0.671~1.922);0.636 | **1.344(1.106~1.634);0.003** |
| **Chronotype** |  |  |
| Morning | Ref. | Ref. |
| Evening | 0.990(0.680~1.443);0.96 | **1.193(1.050~1.354);0.007** |
| **Snoring** |  |  |
| No | Ref. | Ref. |
| Yes | 1.188(0.793~1.781);0.404 | 1.023(0.897~1.168);0.732 |
| **Daytime dozing** |  |  |
| Never/rarely | Ref. | Ref. |
| Occasionally | 0.942(0.597~1.487);0.798 | 1.006(0.866~1.169);0.933 |
| Usually | 1.050(0.332~3.323);0.934 | 1.255(0.875~1.799);0.217 |
| **Note: All models use the fully adjusted Model 3 (adjusted for age, ethnicity, Townsend deprivation index, education, smoking, alcohol, physical activity, diet, medical history, reproductive factors, oral contraceptives, and HRT). p < 0.05 indicates statistically significant results.*  *Abbreviations: HR: Hazard Ratio; CI: Confidence Interval* | | |

**Table S8. Stratified Analysis by Hormone Replacement Therapy (HRT) Use**

| **Characteristic** | **HRT (No)** | **HRT (Yes)** |
| --- | --- | --- |
|  | **HR（95%CI）；*p*** | **HR（95%CI）；*p*** |
| **Group of sleep durations** |  |  |
| 7-9h | Ref. | Ref. |
| ≤6h | **1.335(1.109~1.606);0.002** | 1.108(0.920~1.334);0.281 |
| ≥10h | 1.295(0.690~2.430);0.421 | **1.956(1.232~3.103);0.004** |
| **Insomnia** |  |  |
| Never/rarely | Ref. | Ref. |
| Occasionally | 0.912(0.719~1.156);0.444 | 1.287(0.981~1.687);0.068 |
| Usually | 1.228(0.960~1.569);0.102 | **1.477(1.119~1.948);0.006** |
| **Chronotype** |  |  |
| Morning | Ref. | Ref. |
| Evening | **1.403(1.182~1.666);<0.001** | 1.073(0.905~1.272);0.416 |
| **Snoring** |  |  |
| No | Ref. | Ref. |
| Yes | 1.159(0.965~1.394);0.115 | **1.312(1.105~1.557);0.002** |
| **Daytime dozing** |  |  |
| Never/rarely | Ref. | Ref. |
| Occasionally | 1.005(0.815~1.238);0.965 | 1.063(0.875~1.290);0.539 |
| Usually | 1.095(0.641~1.870);0.741 | 1.382(0.882~2.166);0.158 |
| **Note: All models use the fully adjusted Model 3 (adjusted for age, ethnicity, Townsend deprivation index, education, smoking, alcohol, physical activity, diet, medical history, reproductive factors, oral contraceptives, and HRT). p < 0.05 indicates statistically significant results.*  *Abbreviations: HR: Hazard Ratio; CI: Confidence Interval* | | |

**Table S****9. Stratified Analysis by Ethnicity**

| **Characteristic** | **Ethnicity (White)** | **Ethnicity (Non-White)** |
| --- | --- | --- |
|  | **HR（95%CI）；*p*** | **HR（95%CI）；*p*** |
| **Group of sleep durations** |  |  |
| 7-9h | **Ref.** | **Ref.** |
| ≤6h | **1.245(1.089~1.422);0.001** | 0.640(0.321~1.278);0.206 |
| ≥10h | **1.630(1.109~2.395);0.013** | 2.355(0.550~10.092);0.249 |
| **Insomnia** |  |  |
| Never/rarely | Ref. | Ref. |
| Occasionally | 1.094(0.911~1.314);0.338 | 0.701(0.333~1.473);0.348 |
| Usually | **1.354(1.121~1.634);0.002** | 0.806(0.355~1.826);0.605 |
| **Chronotype** |  |  |
| Morning | Ref. | Ref. |
| Evening | **1.219(1.078~1.378);0.002** | 1.476(0.798~2.731);0.215 |
| **Snoring** |  |  |
| No | Ref. | Ref. |
| Yes | **1.190(1.046~1.353);0.008** | **2.866(1.581~5.198);0.001** |
| **Daytime dozing** |  |  |
| Never/rarely | Ref. | Ref. |
| Occasionally | 1.031(0.891~1.193);0.685 | 1.226(0.646~2.326);0.534 |
| Usually | 1.291(0.907~1.839);0.157 | 1.137(0.264~4.908);0863 |
| **Note: All models use the fully adjusted Model 3 (adjusted for age, ethnicity, Townsend deprivation index, education, smoking, alcohol, physical activity, diet, medical history, reproductive factors, oral contraceptives, and HRT). p < 0.05 indicates statistically significant results.*  *Abbreviations: HR: Hazard Ratio; CI: Confidence Interval* | | |

**Table S10. Stratified by Diabetes History**

| **Characteristic** | **Diabetes mellitus (No)** | **Diabetes mellitus (Yes)** |
| --- | --- | --- |
|  | **HR（95%CI）；*p*** | **HR（95%CI）；*p*** |
| **Group of sleep durations, N (%)** |  |  |
| 7-9h | Ref. | Ref. |
| ≤6h | **1.240(1.085~1.417);0.002** | 0.794(0.399~1.579);0.51 |
| ≥10h | **1.671(1.137~2.456);0.009** | 1.329(0.315~5.605);0.699 |
| **Insomnia** |  |  |
| Never/rarely | Ref. | Ref. |
| Occasionally | 1.040(0.868~1.245);0.674 | 2.160(0.755~6.180);0.151 |
| Usually | **1.299(1.079~1.564);0.006** | 2.119(0.714~6.287);0.176 |
| **Chronotype** |  |  |
| Morning | Ref. | Ref. |
| Evening | **1.211(1.071~1.370);0.002** | 1.562(0.896~2.721);0.116 |
| **Snoring** |  |  |
| No | Ref. | Ref. |
| Yes | **1.221(1.074~1.389);0.002** | 1.587(0.902~2.795);0.109 |
| **Daytime dozing** |  |  |
| Never/rarely | Ref. | Ref. |
| Occasionally | 1.025(0.885~1.187);0.741 | 1.232(0.679~2.235);0.492 |
| Usually | 1.248(0.871~1.788);0.227 | 1.363(0.401~4.634);0.62 |
| **Note: All models use the fully adjusted Model 3 (adjusted for age, ethnicity, Townsend deprivation index, education, smoking, alcohol, physical activity, diet, medical history, reproductive factors, oral contraceptives, and HRT). p < 0.05 indicates statistically significant results.*  *Abbreviations: HR: Hazard Ratio; CI: Confidence Interval* | | |

**Table S11. Stratified by Hypertension History**

| **Characteristic** | **Hypertension (No)** | **Hypertension (Yes)** |
| --- | --- | --- |
|  | **HR（95%CI）；*p*** | **HR（95%CI）；*p*** |
| **Group of sleep durations, N (%)** |  |  |
| 7-9h | Ref. | Ref. |
| ≤6h | 1.187(1.004~1.403);0.045 | **1.258(1.019~1.554);0.033** |
| ≥10h | **1.648(1.000~2.716);0.05** | 1.646(0.942~2.874);0.08 |
| **Insomnia** |  |  |
| Never/rarely | Ref. | Ref. |
| Occasionally | **1.142(0.918~1.422);0.233** | 0.943(0.696~1.277);0.704 |
| Usually | **1.318(1.049~1.656);0.018** | 1.301(0.959~1.767);0.091 |
| **Chronotype** |  |  |
| Morning | Ref. | Ref. |
| Evening | 1.126(0.966~1.312);0.129 | **1.410(1.159~1.714);0.001** |
| **Snoring** |  |  |
| No | Ref. | Ref. |
| Yes | **1.255(1.069~1.473);0.005** | 1.202(0.982~1.470);0.074 |
| **Daytime dozing** |  |  |
| Never/rarely | Ref. | Ref. |
| Occasionally | 1.056(0.880~1.268);0.559 | 1.005(0.801~1.260);0.969 |
| Usually | 1.404(0.907~2.174);0.128 | 1.065(0.610~1.861);0.824 |
| **Note: All models use the fully adjusted Model 3 (adjusted for age, ethnicity, Townsend deprivation index, education, smoking, alcohol, physical activity, diet, medical history, reproductive factors, oral contraceptives, and HRT). p < 0.05 indicates statistically significant results.*  *Abbreviations: HR: Hazard Ratio; CI: Confidence Interval* | | |

**Table S12. Stratified Analysis by Townsend Deprivation Index (TDI) Tertile**

| **Characteristic** | **TDI (Q1)** | **TDI (Q2)** | **TDI (Q3)** |
| --- | --- | --- | --- |
|  | **HR（95%CI）；*p*** | **HR（95%CI）；*p*** | **HR（95%CI）；*p*** |
| **Group of sleep durations** |  |  |  |
| 7-9h | Ref. | Ref. | Ref. |
| ≤6h | **1.374(1.076~1.755);0.011** | 1.037(0.819~1.314);0.763 | **1.266(1.030~1.557);0.025** |
| ≥10h | 0.678(0.216~2.125);0.504 | **2.073(1.160~3.703);0.014** | **1.837(1.072~3.148);0.027** |
| **Insomnia** |  |  |  |
| Never/rarely | Ref. | Ref. | Ref. |
| Occasionally | 1.213(0.854~1.723);0.282 | 1.155(0.851~1.569);0.355 | 0.922(0.698~1.219);0.569 |
| Usually | **1.550(1.080~2.224);0.017** | 1.253(0.910~1.725);0.166 | 1.241(0.934~1.649);0.137 |
| **Chronotype** |  |  |  |
| Morning | Ref. | Ref. | Ref. |
| Evening | 0.934(0.737~1.185);0.575 | 1.222(0.994~1.503);0.057 | **1.483(1.224~1.798);<0.001** |
| **Snoring** |  |  |  |
| No | Ref. | Ref. | Ref. |
| Yes | **1.379(1.093~1.740);0.007** | 1.003(0.802~1.254);0.977 | **1.341(1.097~1.639);0.004** |
| **Daytime dozing,** |  |  |  |
| Never/rarely | Ref. | Ref. | Ref. |
| Occasionally | 1.111(0.848~1.456);0.443 | 1.041(0.813~1.332);0.751 | 0.984(0.783~1.235);0.888 |
| Usually | 1.456(0.748~2.834);0.269 | 1.564(0.896~2.729);0.116 | 0.973(0.545~1.736);0.925 |
| **Note: All models use the fully adjusted Model 3 (adjusted for age, ethnicity, Townsend deprivation index, education, smoking, alcohol, physical activity, diet, medical history, reproductive factors, oral contraceptives, and HRT). p < 0.05 indicates statistically significant results.*  *Abbreviations: HR: Hazard Ratio; CI: Confidence Interval* | | | |
